# Brain-Computer Interfaces for Communication: Preferences of Individuals With Locked-in Syndrome

**DOI:** 10.1177/1545968321989331

**Published:** 2021-02-03

**Authors:** Mariana P. Branco, Elmar G. M. Pels, Ruben H. Sars, Erik J. Aarnoutse, Nick F. Ramsey, Mariska J. Vansteensel, Femke Nijboer

**Affiliations:** 1 University Medical Center Utrecht, Netherlands; 2 Leiden University, Netherlands; 3 University of Twente, Enschede, Netherlands

**Keywords:** brain-computer interface, communication, opinion, user-centered design, locked-in syndrome

## Abstract

**Background:**

Brain-computer interfaces (BCIs) have been proposed as an assistive technology (AT) allowing people with locked-in syndrome (LIS) to use neural signals to communicate. To design a communication BCI (cBCI) that is fully accepted by the users, their opinion should be taken into consideration during the research and development process.

**Objective:**

We assessed the preferences of prospective cBCI users regarding (1) the applications they would like to control with a cBCI, (2) the mental strategies they would prefer to use to control the cBCI, and (3) when during their clinical trajectory they would like to be informed about AT and cBCIs. Furthermore, we investigated if individuals diagnosed with progressive and sudden onset (SO) disorders differ in their opinion.

**Methods:**

We interviewed 28 Dutch individuals with LIS during a 3-hour home visit using multiple-choice, ranking, and open questions. During the interview, participants were informed about BCIs and the possible mental strategies.

**Results:**

Participants rated (in)direct forms of communication, computer use, and environmental control as the most desired cBCI applications. In addition, active cBCI control strategies were preferred over reactive strategies. Furthermore, individuals with progressive and SO disorders preferred to be informed about AT and cBCIs at the moment they would need it.

**Conclusions:**

We show that individuals diagnosed with progressive and SO disorders preferred, in general, the same applications, mental strategies, and time of information. By collecting the opinion of a large sample of individuals with LIS, this study provides valuable information to stakeholders in cBCI and other AT development.

## Introduction

Stroke, trauma, or neuromuscular diseases (NMDs), such as amyotrophic lateral sclerosis (ALS), are merely a few examples of the causes that can lead to complete loss of voluntary muscle control and subsequent loss of communication. When people have no means to communicate other than eye movements, they are considered to be in a locked-in state (locked-in syndrome [LIS]).^1^ LIS is characterized by aphonia or severe hypophonia, preserved cognition, and quadriplegia or quadriparesis and affects about 0.8 out of 100 000 people in Western Europe.^2^ Contrarily to what is generally expected, individuals with LIS report a satisfactory quality of life, which mostly depends on their ability to communicate and to be autonomous.^3-5^

In the past decades, several communication brain-computer interfaces (cBCIs) have been developed as alternative assistive technology (AT) and have been evaluated with individuals with LIS.^6-11^ To develop functional and widely accepted cBCI technology, the user’s opinion and participation throughout the research and development phases of the AT (user-centered design [UCD]) is crucial.^12^ For that reason, UCD has received increasing attention in the BCI field with the goal to manage BCI users’ expectations and to prevent the abandonment of technology by the end user.^6,13-22^ These studies focused not only on the technical specifications of the BCI system, such as the technique used for recording brain signals (internal or external electrodes^23^) and general BCI applications (what a user would like to control with a BCI and how well it should work; eg, Huggins et al^14,19^), but also on the subjective acceptance of the technology.^16,20^ Although the opinion of prospective users about BCI applications (beyond communication) has been investigated before,^14,19^ to our knowledge, no information has been collected about the user’s opinions on different mental strategies for cBCI control, nor on when in the course of their medical condition users would like to be informed about AT and cBCIs. The latter, especially when the user needs change as a result of progression of the disease, may also affect the acceptance of AT.

In this study, we investigated the opinion of prospective cBCI users in the Netherlands regarding (1) which applications they would like to control with a cBCI, (2) which mental strategies they would prefer to use to control the cBCI, and (3) the time point during their clinical trajectory at which they would like to be informed about ATs, including cBCIs. Importantly, the nature of the underlying medical conditions (ie, progressive disease vs stable condition after an event) may affect the preference of the user with respect to these 3 research questions. Therefore, we compared participants with respect to the type of condition underlying LIS—that is, NMD or sudden onset (SO) events. A better understanding of these preferences is fundamental for an efficient and effective development of cBCIs and for increasing the likelihood of adoption of this technology for autonomous home use.

## Material and Methods

A questionnaire was administered to individuals with LIS who were making use of AT for communication and who could potentially benefit from cBCIs. The questionnaire (in Dutch) was completed by the participant during a 3-hour home visit with 2 researchers. One researcher interviewed the participant while the other researcher made observations and confirmed the answers from the participants. The questionnaire was implemented on the Qualtrics Survey platform (https://www.qualtrics.com/). The study was evaluated by the local ethics board of the Utrecht Medical University Center, who determined it to be exempt from the Medical Scientific Research Act. In accordance with the local ethics guidelines, GCP and the GDPR, at the beginning of the home visit, participants or (when a participant was unable to write) caregivers on behalf of the participant gave written informed consent to participate in the study.

### Participants

A total of 40 participants living in the Netherlands were identified using databases obtained in earlier studies and were invited to participate in this study.^2,18^ Candidates were approached through email and received an information letter about the research. When interested in participating in the study, the candidates were sent an online screening form, which was used for participant characterization and demographics (see Section 1 of the questionnaire). Inclusion criteria in the study were as follows: (1) (in)complete LIS, defined as in American Congress of Rehabilitation Medicine^1^; (2) the ability to indicate yes and no reliably and to thereby give informed consent to participate in the study; and (3) the ability to answer open questions, either with the help of a letter card or through an AT device. Eligible candidates were subsequently contacted by a researcher to plan a 3-hour home visit. In total, 29 participants enrolled in the study (73% response rate; BQ1-BQ29). One participant (BQ4) was excluded from further analysis after the home visit because of unreliable means of communication, yielding 28 complete interviews (Supplementary Table 1). Of these, 2 participants were implanted with a cBCI at the time of the interview.^9,24^

The revised Amyotrophic Lateral Sclerosis Functional Rating Scale (ALSFRS-r^25^) score was used to assess the level of paralysis and communication impairment of each participant. Responses to each item of the ALSFRS-r were described by the participant and caregiver and scored by both researchers independently. For comparison purposes, the participant population was divided by type of disorder into one group with neuromuscular disorder (such as ALS, primary lateral sclerosis, and progressive spinal muscular atrophy) and another group with SO (such as trauma or stroke). There were in total 13 NMD and 15 SO participants.

### Structure of the Questionnaire

**Figure 1.**
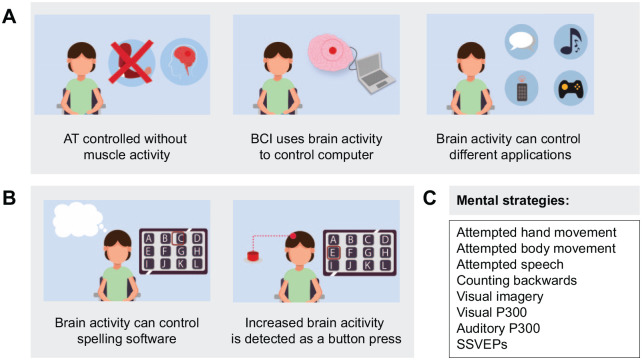
Representative screenshots of the animation videos used in the questionnaire. A total of 9 animations videos were shown to each participant. A. Section 2 of the questionnaire used an animation video to introduce communication brain-computer interfaces (cBCIs). Three illustrative screenshots of the video explaining what a cBCI is, how to control it, and what can it be used for are shown. In this video, we described the concept of an ideal cBCI that would be 100% accurate and 100% accepted by the users. B. In section 4 of the questionnaire, animation videos were presented to the participants, each describing a different mental strategy. For simplicity, consistency across mental strategies, and to avoid the application biasing the mental strategy, all videos showed a spelling matrix as a control application (left screenshot) and a button press (and subsequent letter selection) as a control output (right screenshot). C. Eight mental strategies were described in the questionnaire: attempted hand movement, attempted body movement (other than hand), attempted speech, counting backward, visual imagery, visual P300, auditory P300, and steady-state visual evoked potentials (SSVEPs). Abbreviation: AT, assistive technology.

The questionnaire consisted of 5 sections: (1) demographics, (2) introduction to cBCIs, (3) cBCI applications, (4) mental strategies, and (5) time of information. Animation videos were used to illustrate certain aspects of the questionnaire, more specifically to introduce the concept of cBCIs (Figure 1A) and each individual mental strategy (Figure 1B). The animations were specifically designed for the purpose of this questionnaire and were narrated in Dutch. The majority of the questions were either multiple-choice or based on a 5-point Likert scale. Participants always had the opportunity to make remarks or to skip a question. Family members/caregivers were often present during the interview but were asked not to answer questions on behalf of the participant. Each question-and-answer option was read out aloud by the researcher. When an animation video was part of the question, the question and answers were read first, after which the video was shown, and then the question and answers were repeated. The answers were only registered once the participant clearly understood the question and the answer options. Finally, at the end of the questionnaire, we asked the participants to rate their willingness to consider a cBCI for communication using a 5-point Likert scale (1 = *very unlikely*, 5 = *very likely*). Ranking questions, applications, and mental strategies were randomly presented to prevent an order effect.

#### Section 1 (Demographics)

Prior to the home visit, participants were asked to fill out an online questionnaire on their demographic information. Questions were either multiple-choice, yes/no, or required a brief open answer. When applicable, more than 1 choice could be selected and an item “Other” was available (followed by a free-text field) to accommodate other options.

#### Section 2 (Introduction)

An animation video (see Figure 1A for examples of snapshots) was used to introduce the concept of BCIs in general and in particular BCIs for communication (cBCIs). In this video, we described the concept of an ideal cBCI that would be 100% accurate and 100% accepted by the users, and we did not provide details about the different signal acquisition, processing, and classification methods. This choice allowed participants to focus on the choice of applications and mental strategies rather than factors such as efficacy, speed, or level of invasiveness.

#### Section 3 (cBCI Applications)

In this section, we asked questions about the AT aids the participants used at the time of the visit at home for communication, what applications they controlled with it, and how often they used that functionality. Additionally, we asked them to rank their preference with respect to 6 different cBCI applications: *private conversation and writing* (eg, email, chat, diary), *direct personal communication* (eg, voice synthesis, direct conversation), *environmental control* (eg, home appliances, alarm), *general computer use* (eg, playing games, internet surfing, social media), *artistic expression* (eg, painting, making music), and *emotions and facial expressions* (eg, expressing feelings, emojis). In this questionnaire, we asked participants about their current AT applications and did not ask about which applications they would like to be offered by a cBCI, because the answer would likely be “all applications.” However, we did ask the participants to rank the application to be provided by a cBCI in order of preference.

#### Section 4 (Mental Strategies)

We assessed the opinion of the participants regarding 8 widely known mental strategies that can be used to generate brain signal changes for cBCIs^26-30^—namely, *attempted hand movement*, *attempted body movement* (other than hand), *attempted speech*, *counting backward*, *visual imagery*, *visual P300*, *auditory P300*, and *steady-state visual-evoked potentials* (SSVEPs; Figure 1C). Each strategy was presented (in random order) using a dedicated animation video (see Figure 1B for examples of snapshots). Of note, to keep the explanation of all strategies consistent, all were illustrated using the same output control: a 1-dimensional control of a button press that selects a letter on a spelling device (Figure 1B), thereby aiming to focus on the concept behind the mental strategy and not the type, speed, or accuracy of the system. Participants were asked to imagine using a perfectly working cBCI. After each animation, the participant was asked to imagine using that particular strategy for about 10 s and then rate it (using a 5-point Likert scale) on clarity, difficulty, and enjoyability (ie, how much fun it was to perform the strategy) and, finally, rank all strategies in order of preference.

#### Section 5 (Time of Information)

In the last section of the questionnaire, we asked the participants’ opinion about the best period to be informed about AT aids in general, including cBCIs. The participants could indicate at what time point during their clinical trajectory they would prefer to have received detailed information about communication aids. Answer options were as follows: “as soon as possible” after diagnosis/incident, “before rehabilitation” (possible period between incident/hospitalization and start of, for example, speech-language therapy, ergotherapy, or other), “during rehabilitation,” “after rehabilitation,” “when no residual movement/speech is available,” or an open field for another time point.

### Data Analysis

#### Descriptive Statistics and Open Answers

Descriptive statistics were used to analyze the survey results. Frequencies, percentages, or medians were used when appropriate. The percentages were computed relative to the total number of participants (ie, n = 28) or, when applicable, to a subgroup of participants.

#### Ranking Questions

To quantify the preference of the participants for specific applications and mental strategies (ranking questions), we attributed a weight to each option between 0 and 6 for the applications and between 0 and 4 for the mental strategies, where 0 represented *not chosen*, 1, *the least preferred*, and 4 or 6, *the most preferred*. The different maximum weights related to the number of items ranked. Notably, the participants ranked all 6 applications because it can be assumed that all applications are (eventually) useful for the user. In contrast, users typically only need one or a few mental strategies to control a cBCI and were, therefore, asked to rank the top 4 (out of 8) mental strategies. Subsequently, we computed the center-of-mass (COM) score previously used in questionnaire analysis^31^ in order to combine ranking scores across participants and to facilitate comparison between options. The COM score was computed per application or mental strategy by summing the product of the number of participants who assigned a certain rank to an application/mental strategy and the weight given to that rank, and subsequently dividing this sum by the total number of participants:

(1) $${\mathrm{COM}}_{i}=\frac{\sum_{k=1}^{N}k\cdot \sum_{s=1}^{T}[{\mathrm{Weight}}_{s}=k]}{T},$$

where *i* represents an application or a mental strategy, *T* the total number of participants (indexed by *s*), *k* the weight between 1 and *N* (ie, the total number of ranked applications, *N* = 6, or mental strategies, *N* = 4), and [Weight_*s*_ = *k*] is 1 if Weight_*s*_ is equal to *k* and 0 otherwise. The COM score varied between 0 and the total number of ranked options (4 or 6), and a larger COM value indicates a more preferred option. Statistical comparison between COM scores is not possible because there is only 1 value per class. Nevertheless, meaningful differences of COM values between applications and mental strategies were computed by applying a Monte Carlo randomization method, where the ranking scores of each participant were assigned randomly 1000 times. The expected chance variance was computed as the SD of the resulting COM distribution. Differences between COM values larger than the expected variance were deemed meaningful.

#### Willingness to Consider cBCI After the Questionnaire

We tested whether there was a relation between the current number of residual movements a participant had at the time of the home visit and their willingness to consider a cBCI after the home visit. The residual movements were grouped as eyes, mouth/head, hand/arm, leg/feet/toes, and residual speech. If the participant had one or more forms of residual movement pertaining to one of the groups, it would count as 1, otherwise 0. The total number of residual movements varied between 0 (*no residual movement at all*) and 5 (*able to control at least 1 form of movement per group*). Linear regression was computed between these factors for both the NMD and SO groups to assess if there was a different tendency between the 2 groups. The coefficient of correlation (*r*^2^) for each regression model is reported.

## Results

### Demographics

**Figure 2.**
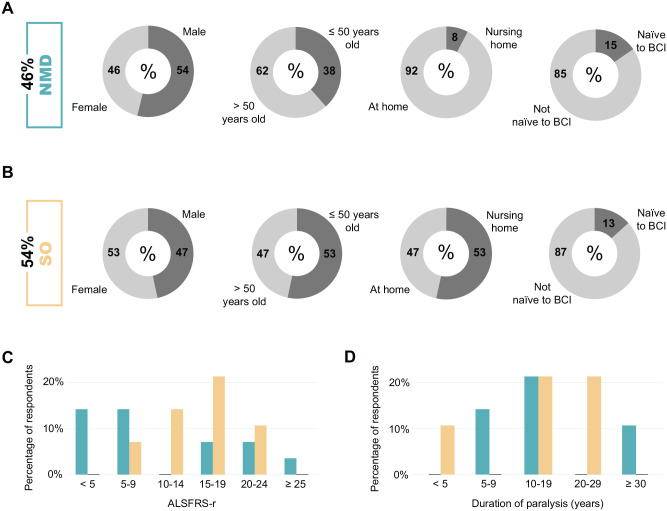
Demographic description of the participants: The demographic information of the participants (n = 28) in this study was extracted from section 1 of the questionnaire. The participants were divided into 2 groups based on the cause of locked-in syndrome—namely, neuromuscular disease (NMD) or sudden onset (SO; A-B) Information (in percentage) about the participants’ gender (male, female), age group (≤50 years old, >50 years old), living situation (at home or in a nursing home), and whether they were naïve to brain-computer interfaces (BCIs) is given for the NMD group (A) and the SO group (B). NMD accounted for 46% (n = 13) of the participants. (C-D) Histogram (in percentage) of the revised Amyotrophic Lateral Sclerosis Functional Rating Scale (ALSFRS-r) score (C) and duration of paralysis in years (D) per group (NMD in green and SO in yellow). Of note, at the time of diagnosis, patients with NMD are often still able to move and speak to a certain extent, hence the exact timing of becoming locked-in (and, therefore, the duration of the locked-in state) is unknown for these participants.

We analyzed responses from 28 participants (median age 53 years; range 29 to 76 years; 14 male). Of these, 13 (46%) were diagnosed with a progressive NMD (Figure 2A), whereas 15 (54%, Figure 2B) had a SO event that led to the locked-in state (see also Supplementary Table 1). In both groups, the number of male and female participants was similar. The majority of the NMD participants (62%) were older than 50 years, lived at home (92%), and had heard of or seen a BCI system in the past (85%). In contrast, the majority of the SO participants (53%) were younger than 50 years old and lived in a nursing home (53%). Similar to the NMD group, the majority of the SO participants (87%) were familiar with the concept of a BCI.

Participants scored a median ALSFRS-r of 15 (on a scale from 0 to 48; Figure 2C), and there was no significant difference in the ALSFRS-r scores between NMD and SO groups (NMD median of 8, range 2-29; SO median of 16, range 8-22; unpaired 2-samples Wilcoxon test, *z* = −0.14, rank sum = 185; NS). The overall median duration since diagnosis was 16.5 years (Figure 2D) and was not significantly different between NMD and SO groups (NMD median of 14.3 years, range 6.3-49.4 years; SO median 16.8 years, range 2.7-29.5 years; unpaired 2-samples Wilcoxon test, *z* = −1.38, rank sum = 158; NS). Of note, the duration since diagnosis only corresponded with the exact duration of LIS for the SO group because individuals with NMD generally entered LIS several months to years after the first diagnosis.

### Residual Movement and Current Communication Channels

**Figure 3.**
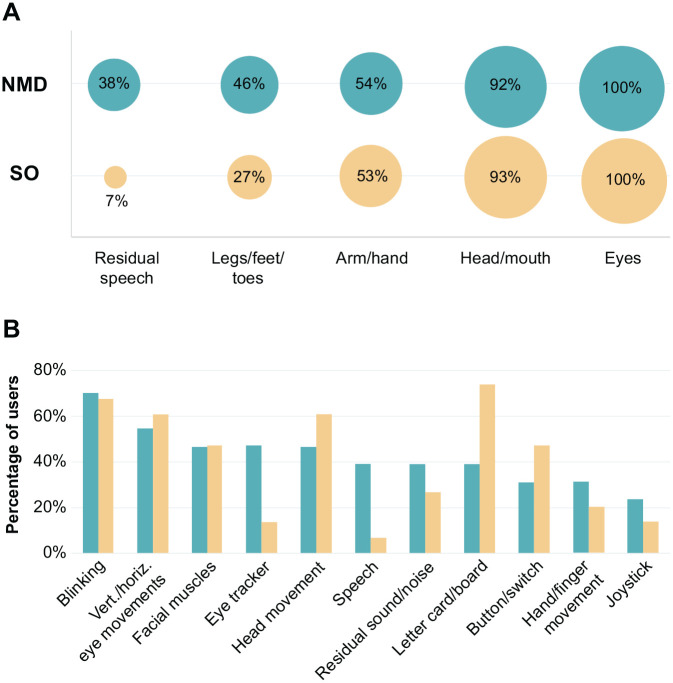
Current communication channels and residual movement: A. Information (in percentage) about the remaining residual movement of the participants per group (neuromuscular disease [NMD], in green; sudden onset [SO], in yellow) at the time of the questionnaire. B. Histogram (in percentage) of the currently used communication channels per group (NMD in green, and SO in yellow). Abbreviation: LIS, locked-in syndrome.

All participants had aphonia or severe hypophonia, and quadriplegia or quadriparesis. Regarding residual movement (Figure 3A), all participants (for both NMD and SO groups) had preserved sustained eye opening and eye movements. The majority of the participants had residual movement of the head or mouth (92% and 93% for NMD and SO, respectively) and/or residual movement of the arm or hand/fingers (54% and 53% for NMD and SO, respectively). However, the NMD group showed higher counts of preserved leg, foot, or toe movement (46%) and residual forms of speech (eg, making noises/sounds or saying short words; 38%) when compared with the SO group (27% and 7%, respectively). The participants reported using a variety of current communication channels (Figure 3B), from eye movements to answer closed questions, to letter cards and more sophisticated hardware switches and eye trackers. Interestingly, letter cards, button switches, and head movements were more used by the SO group than the NMD group. In contrast, NMD participants used eye trackers and residual forms of speech more than the SO group. The latter is likely related to the fact that the NMD group had considerably more individuals with preserved forms of residual speech compared with the SO group (38% against 7%).

### Preferred Applications

**Figure 4.**
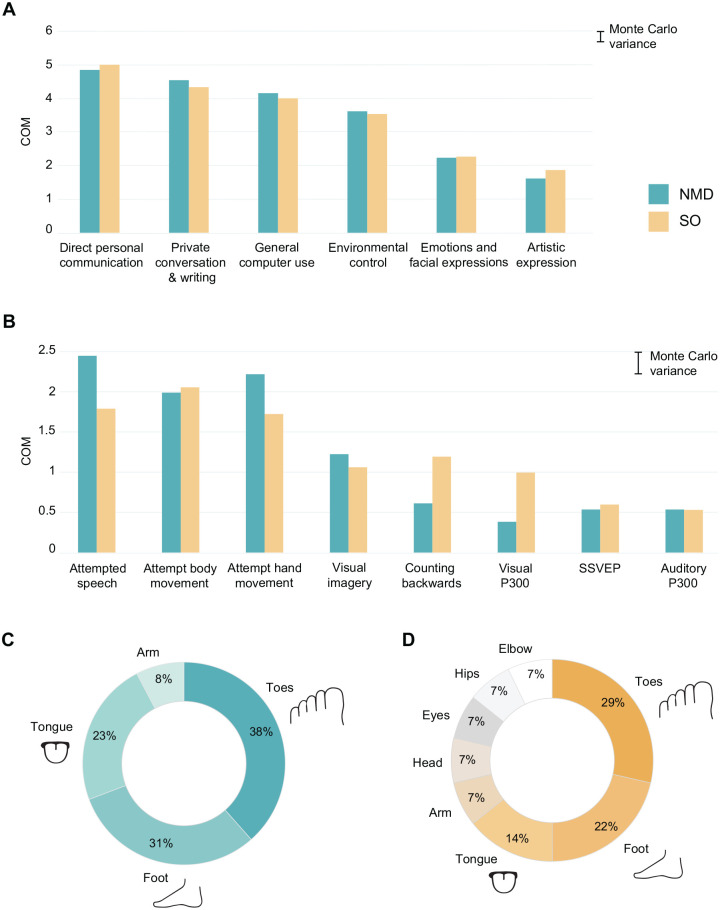
Preferred applications and mental strategies: (A-B) Ranking (using the center-of-mass metric, COM) of preferred applications (A) and mental strategies (B) possibly supported by a cBCI, by individuals with neuromuscular disease (NMD; n = 13, in green) and sudden onset disease (SO; n = 15, in yellow). Statistical difference between bars can be evaluated using the Monte Carlo variance indicated on the top right corner: 0.34 in (A) and 0.28 in (B). (C-D) Body parts (other than hand) selected by the participants during the “attempted body movement” strategy for both the NMD (C) and SO (D) groups. Rating scales ranged from 1 (*least preferred*) to 6 (*most preferred*) for A, and from 1 (*least preferred*) to 4 (*most preferred*) for B. Abbreviations: cBCI, communication brain-computer interface; SSVEP, steady-state visual-evoked potential.

Participants considered “direct personal communication” the most important application to be provided by a cBCI, followed by “private conversation and writing” (Figure 4A). “General computer use” came third, followed by “environmental control.” “Emotions and facial expressions” and “artistic expression” were the 2 least preferred applications, being meaningfully inferior to the top 4 applications (difference larger than Monte Carlo variance 0.34). Although the SO group seemed to slightly prefer “artistic expression” (eg, painting or making music) when compared with the NMD group, there was no meaningful difference between the NMD and SO group ratings for any application. Participants were allowed to suggest other applications besides the ones included in this questionnaire. Most of them gave specific examples of environmental control (such as controlling a wheelchair, doors, or DVD player) and of specific computer programs (such as a text editor, e-books, and games). In addition, smartphone control (eg, for video calls), speech synthesis, and alarm functionality were mentioned.

### Preferred Mental Strategies

Attempted body movements (attempted speech, attempted hand movement, and attempted body movement) were the top-rated mental strategies, whereas counting backward, visual P300, SSVEPs, and auditory P300 were the least preferred strategies (Figure 4B). Interestingly, the NMD group rated attempted speech and attempted hand movement meaningfully higher than the SO group (difference larger than the Monte Carlo variance 0.28). In contrast, the SO group rated counting backward and visual P300 higher than the NMD group.

Regarding attempted body movement, we asked participants which body part they would choose to control a cBCI (other than the hand). Toes (38% and 29% for NDM and SO, respectively) and feet (31% and 22% for NDM and SO, respectively) were the most chosen body parts (Figure 4C-4D). Tongue was the third most chosen body part by both groups. Whereas the SO group suggested more alternative body parts (arm, head, eyes, hips, and elbow) to toes and feet, the NMD group only suggested 1 additional body part (arm). When asked to suggest other mental strategies, the participants suggested visual letter imagination, laughing, thinking of colors, tastes/smells, and walking as mental strategies.

### Time of Information

**Figure 5.**
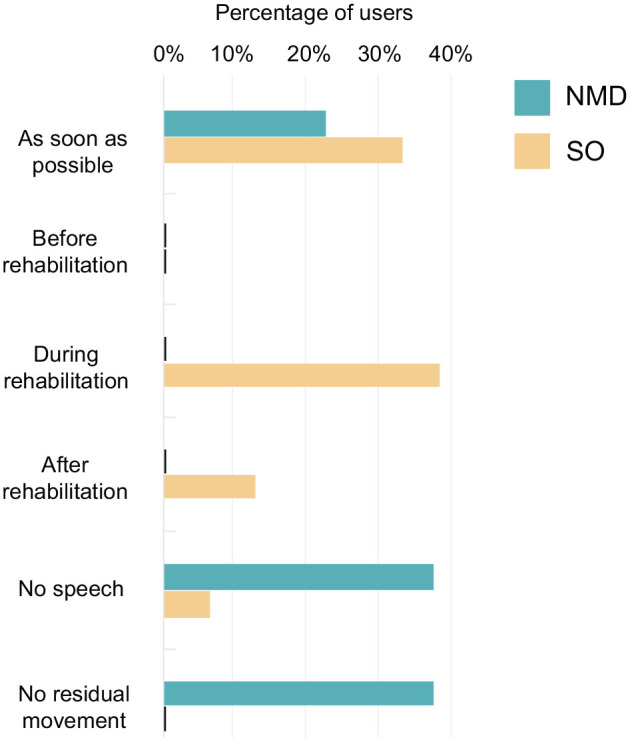
Time of information: Percentage of participants per group (neuromuscular diseases [NMD], in green; sudden onset [SO], in yellow) who would like to be informed about AT solutions (including cBCIs) in different phases after the onset or during the disease progression. Abbreviations: AT, assistive technology; cBCI, communication brain-computer interface.

Both groups preferred to learn about and receive ATs when they approached or entered the locked-in state (Figure 5). Whereas for the NMD group that moment coincides with later stages of their disease (eg, no speech and residual movement), for the SO group, that is immediately after onset or during the rehabilitation phase.

### Willingness to Consider cBCIs

**Figure 6.**
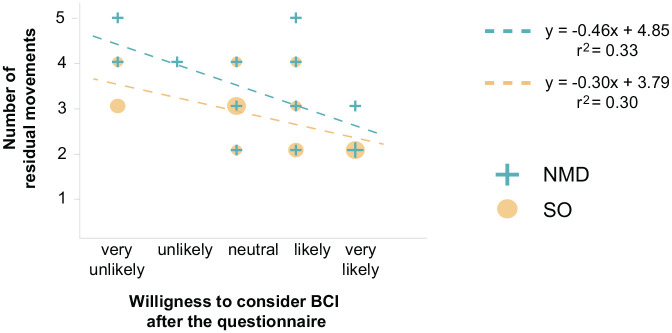
Willingness to consider a communication brain-computer interface (cBCI): Linear regression (dashed lines) between the number of residual movements the participant had at the time of the survey (from 1 to 5; see Figure 3A) and the willingness to consider a cBCI after the end of the questionnaire (from *very unlikely* to *very likely* on a 5-point scale). NMD participants are indicated with a green cross and SO participants with a yellow circle. The size of the cross/circle indicates the number of participants per coordinate: that is, the larger the cross/circle, the more the number of participants. The linear regression equation and corresponding *r*^2^ (per group) are indicated on the top right corner.

Lower number of residual movements correlated moderately with an increased likelihood to consider a cBCI for both NMD and SO groups (*r*^2^_NMD_ = 0.33, *r*^2^_SO_ = 0.30; Figure 6). When explaining their answer, the participants, most of whom had previous knowledge or experience with cBCI technology, considered current BCIs to have limited benefit over current ATs. The majority of these participants reported that they would consider cBCI if “the speed would match current AT” or when the cBCI would be “faster, have equivalent applications [to current ATs] and be esthetically good.” Yet most participants did state that if residual movement/function deteriorates with time, they would consider a cBCI as an option.

## Discussion

In this study, we gathered the opinion of Dutch individuals with LIS regarding 3 aspects of cBCIs: the applications to control with a cBCI, the mental strategies used to generate the control signal, and the moment they would like to be informed about cBCIs. We grouped the participants into NMD and SO and show that preferences of both groups on these aspects correspond to a large extent.

### Preferred Applications

In this study, we show that the choice of cBCI applications does not depend on the cause of LIS. Direct personal communication, private conversation, and general computer use were the top 3 applications chosen by both groups. This is in agreement with earlier studies that investigated quality of life in LIS and that reported a strong relation between quality of life and the ability to communicate.^3-5^ Regarding environmental control, the fourth most chosen application, the participants expressed their desire to control lights, doors, wheelchairs, and DVD players, which is also in correspondence with a previous survey performed by Huggins et al^14^ among individuals with ALS. Interestingly, in contrast to previous studies that suggested that artistic expression is an important BCI tool for several individuals with severe motor paralysis,^32-36^ we found that this application was one of the least preferred. In the current study, artistic expression may have received a lower rank because (as in the general population) not all individuals are actively involved in artistic expression and such application is not provided by all current communication aids, whereas communication is a universally used concept.

### Preferred Mental Strategies

Both SO and NMD participants had a strong preference for motor strategies (attempted speech and movement) over working memory and reactive (evoked) strategies (ie, visual P300, SSVEPs, and auditory P300). This result demonstrates that individuals with LIS prefer to use an active rather than a reactive strategy for cBCI control. The somewhat lower preference of SO participants, compared with NMD participants, for motor-related paradigms could be related to earlier observations in LIS individuals with pontine, premotor, and parietal lesions, who were selectively impaired in mental manipulation of, for example, the hands.^37-40^ Interestingly, one participant, who was paralyzed from birth as a result of cerebral palsy, said that she “cannot imagine or attempt to make a movement.” This is in agreement with previous BCI studies with individuals with CP (eg, Daly et al^41,42^) and with studies that showed that people with fetal brain damage may experience more difficulty acquiring reliable control of their motor functions (eg, Palisano et al^43^).

Surprisingly, apart from the hand, toes and feet were the most chosen body parts for cBCI control, even though only 6 (out of 9 selecting toes/feet) NMD and 2 (out of 7 selecting toes/feet) SO participants could still move their toes and/or feet to a certain extent. Another explanation could be the fact that feet are large body parts of which dexterity is evolutionarily close to that of the hand^44-46^ and, therefore, easy to imagine/attempt control. Nevertheless, a biased choice for these body parts cannot be completely ruled out because feet and toes were the first and the last examples of 4 body parts shown in the animation video (“foot, head, tongue, or toes”). This psychological effect is commonly known as the serial-position effect, where a person tends to recall the first and last items in a sequence best.^47^

Taken together, both SO and NMD groups largely agree on preferred mental strategies for cBCI control. However, the relative stronger preference of the NMD group for attempted speech and hand movement and of the SO group for counting backward and visual P300 overtly highlights the need to consider users’ preference for strategies in future research on BCIs for communication. Moreover, further investigation on the relation between the mental strategy preference and the actual cBCI performance using that strategy may be the key to detect the best features for accurate and stable cBCI control.

### Willingness to Consider cBCI

With fewer residual movements available, participants were more likely to consider a cBCI after the survey. This trend was slightly more pronounced for the NMD groups, which is not surprising because these individuals are more likely to lose their current residual^48^ movements with the progression of the disease. However, the low effect size indicates that a larger number of participants is required to corroborate these findings. Most of the participants indicated that the current BCIs had limited benefit compared with current available AT. As expected, many participants would consider a cBCI if its speed, accuracy, and/or appearance (with respect to noninvasive setups) could be improved over that of currently available systems. Some participants also expressed their thought regarding the type of BCI systems currently available. External (noninvasive) cBCIs were, in general, preferred over internal (implanted) cBCIs (8 against 2, out of 28). Of the 10 participants who expressed their opinion, 2 NMD participants were open to the possibility of an internal cBCI provided that a short hospitalization would be required, which supports previous studies that reported the strong preference of users for outpatient surgery.^14,19,23^

### Time of Information

Another topic that has received little attention in the field of BCI is the time of information—that is, the preferred moment when users would like to be informed about and try cBCIs as a communication AT aid. In this study, we investigated this subject and found that both NMD and SO groups have a stronger preference to be informed when they most need an AT—that is, when they reach the locked-in state. Naturally, this yields different time points in the clinical trajectory of the NMD and SO groups because patients with progressive disorders have (or perhaps need) more time to accept their situation and generally only require AT sometime after diagnosis, whereas individuals with SO disorders are often faced with immediate LIS and need AT aids directly. Yet some NMD participants also expressed their wish to be informed as soon as possible after diagnosis. This dichotomic result is in line with the findings of a recent qualitative study on veterans with ALS, which reports that whereas many patients described the urgency to be proactive with respect to BCIs before they lose muscular control and verbal communication, others prefer to wait for eventual new technology and to enjoy their remaining time with their family.^48^ Altogether, our results reinforce the idea that the BCI community should play a more active role in informing rehabilitation centers about cBCIs, such that these can be tested by SO users shortly after the event.

### Strengths and Limitations

In this pioneer study, we interviewed 28 Dutch individuals with LIS, which covers an ample portion of the LIS population in the Netherlands (a total estimated number of 124 patients at a prevalence of 0.73 patients per 100 000, (Pels, Aarnoutse, Ramsey and Vansteensel, 2017)). In this pooled sample, the median duration of paralysis was 16.5 years, indicating that individuals with LIS in the Netherlands live many years with this condition, which deepens the value of their opinion on cBCIs. Another strength of this study was the delivery of a questionnaire through structured interviews at the participant’s home. During the home visits, we had the opportunity to explain the questions and collect the participants’ opinions with respect to several open questions at their own pace. Furthermore, for the purpose of this study, we designed and validated several animation videos that introduce and explain several concepts related to cBCIs.

This study also has some limitations. In the past years, a number of studies related to BCI have been conducted in the Netherlands and included some of the current participants. As a result, a majority of the participants (86%) were not naïve to the concept of BCI before the questionnaire described here. Although this fact may have influenced our results, we explained to the participants during the home visits that this questionnaire was about an ideal cBCI and not the ones they had experimented with in the past (if that was the case). Often caregivers and family members were present during the user interviews. Even though they were asked not to answer on behalf of the user, their presence could have theoretically biased the preferences of the users.

Regarding the mental strategies, it cannot be ruled out that some participants have comorbid cognitive problems that make it difficult for them to use certain strategies. An answer to this question would require access to medical information or a full evaluation of their cognitive capabilities, which was beyond the scope of this study. However, the participants’ communication during the home visits did not lead to any suspicion of impaired cognition. Also, the terms used in this study to describe the time of information were not always applicable to both the NMD and SO groups. Nevertheless, we believe that the use of the respective terms is unavoidable considering the different natures of the participants’ clinical conditions. Finally, because of the (still) limited sample size, advanced regressive models were not applied. A larger (and international) cohort would be required to statistically assess the relation between the ranked applications and strategies and observed variables such as age, duration of paralysis, and ALSFRS-r scores. We make the methods in this study openly available to allow other researchers to run this questionnaire in their user cohort.

## Conclusion

In this study, we investigated the opinion of prospective cBCI users regarding 2 important aspects of BCIs for communication: the mental strategies for control and the controlled output application. We showed that individuals with LIS consider (in)direct communication, general computer use, and environmental control to be important features of a cBCI and that attempted speech and movement as control strategies are preferred over reactive strategies, such as P300 and SSVEPs. Moreover, the preferred time to be informed about AT aids and cBCI is when the user reaches the locked-in state and needs the AT. We believe that this survey provides valuable information to stakeholders in cBCI and AT development and encourages the involvement of users in the research and development process, ultimately promoting an optimal cBCI design and reducing risk of technology abandonment.

## Supplemental Material

sj-pdf-1-nnr-10.1177_1545968321989331 – Supplemental material for Brain-Computer Interfaces for Communication: Preferences of Individuals With Locked-in Syndrome Click here for additional data file. Supplemental material, sj-pdf-1-nnr-10.1177_1545968321989331 for Brain-Computer Interfaces for Communication: Preferences of Individuals With Locked-in Syndrome by Mariana P. Branco, Elmar G. M. Pels, Ruben H. Sars, Erik J. Aarnoutse, Nick F. Ramsey, Mariska J. Vansteensel and Femke Nijboer in Neurorehabilitation and Neural Repair
