# Supplementary material for: Brain-Computer Interfaces for Communication: Preferences of Individuals With Locked-in Syndrome
Source: Neurorehabil Neural Repair. 2021 Feb 3;35(3):267–79. doi: 10.1177/1545968321989331 (PMC7934157; doi:10.1177/1545968321989331)
Supplement: sj-pdf-1-nnr-10.1177_1545968321989331 – Supplemental material for Brain-Computer Interfaces for Communication: Preferences of Individuals With Locked-in Syndrome [file sj-pdf-1-nnr-10.1177_1545968321989331.pdf]

Supplementary Material

Supplementary Table 1 – Participant demographics: age (years), gender (female, F; male, M), disorder (Amyotrophic Lateral Sclerosis, ALS; Primary Lateral Sclerosis, PLS; Progressive Spinal Muscular Atrophy, PSMA; Spinal Muscular Atrophy, SMA); ALSFRS-r (Amyotrophic Lateral Sclerosis Functional Rating Scale revised) score, ranging from 0 to 48, where 48 denotes no impairment; duration since diagnosis (in years); and number of communication channels available at the time of the home-visit. Of note, at the time of diagnosis, patients with NMD are often still able to move and speak to a certain extent, hence the exact timing of becoming locked-in (and therefore the duration of the locked-in state) is unknown for these participants.

Participant	Age	Sex	Etiology	ALSFRS-r	Duration since diagnosis (in years)	Number of communication channels
**Neuromuscular Diseases (NMD)**						
BQ01	61	F	ALS	1	9.9	4
BQ02	49	F	ALS	3	14.8	6
BQ03	47	F	ALS	17	6.3	6
BQ06	60	M	PLS	29	14.3	2
BQ08	54	M	PSMA	7	9.1	4
BQ09	58	F	ALS	3	12.3	6
BQ11	57	M	ALS	22	8.3	6
BQ13	57	F	ALS	23	19.0	2
BQ16	49	M	SMA	7	49.4	7
BQ17	42	F	SMA	9	42.2	1
BQ22	66	M	ALS	4	18.2	6
BQ26	38	M	SMA	8	38.3	9
BQ27	70	M	ALS	19	12.7	1
**Sudden Onset (SO)**						
BQ05	29	F	Cerebral Palsy	16	29.5	8
BQ07	34	F	Ischemic stroke	22	16.3	5
BQ10	60	M	Brain stem stroke	9	3.6	5
BQ12	49	F	Brain stem stroke	18	2.9	3
BQ14	46	F	Ischemic stroke	22	28.5	10
BQ15	76	F	Ischemic stroke	22	26.0	1
BQ18	54	M	Brain stem stroke	8	26.4	2
BQ19	46	M	Ischemic stroke	14	10.0	4
BQ20	40	F	Brain stem stroke	17	11.7	3
BQ21	37	M	Traumatic brain injury followed by Meningitis	18	16.8	1
BQ23	56	M	Traumatic brain injury	12	26.3	4
BQ24	63	F	Brain stem stroke	14	16.7	8
BQ25	57	M	Embolism	16	18.8	3
BQ28	44	M	Ischemic stroke	10	2.7	4
BQ29	52	F	Brain stem stroke	16	20.3	4
